# Are horses capable of mirror self-recognition? A pilot study

**DOI:** 10.1371/journal.pone.0176717

**Published:** 2017-05-16

**Authors:** Paolo Baragli, Elisa Demuru, Chiara Scopa, Elisabetta Palagi

**Affiliations:** 1 Dipartimento di Scienze Veterinarie, Università di Pisa, Pisa, Italy; 2 Museo di Storia Naturale, Università di Pisa, Calci, Italy; 3 Unità di Primatologia Cognitiva, ISTC-CNR, Roma, Italy; University of Rennes 1, FRANCE

## Abstract

Mirror Self-Recognition (MSR) unveils complex cognitive, social and emotional skills and it has been found only in humans and few other species, such as great apes, dolphins, elephants and magpies. In this pilot study, we tested if horses show the capacity of MSR. Four subjects living socially under naturalistic conditions were selected for the experiment. We adopted the classical mark test, which consists in placing a coloured mark on an out-of-view body part, visible only through mirror inspection. If the animal considers the image as its own, it will use its reflection to detect the mark and will try to explore it. We enhanced the classical paradigm by introducing a double-check control. Only in the presence of the reflecting surface, animals performed tactile and olfactory exploration of the mirror and looked behind it. These behaviors suggest that subjects were trying to associate multiple sensory cues (visual, tactile and olfactory) to the image in the mirror. The lack of correspondence between the collected stimuli in front of the mirror and the response to the colored mark lead us to affirm that horses are able to perceive that the reflected image is incongruent when compared with the memorized information of a real horse. However, without replication of data, the self-directed behavior towards the colored marks showed by our horses cannot be sufficient *per se* to affirm that horses are capable of self-recognition.

## Introduction

The earliest evidence of horse domestication traces back to about 6,000 years ago [1]. Since then, horses were mostly used as working animals and, in the latest centuries, they also became one of our preferred domesticated animals, developing a billionaire trade all over the world [2]. The special bond linking horses and humans is also witnessed by the important role they play in Animal-Assisted Interventions [3,4] and some recent findings suggest that physiological variables (e.g., heart rate variability) of humans and horses can show a sort of coupling process that is modulated by the kind and time duration of the contact interaction they engage in [5]. Such economic and social impact has stimulated the scientific interest regarding behavior and cognition of horses. Recent findings suggest that horses are able to solve three-choice tasks in a flexible way [6], to use their long-term memory for concept and categories [7], to integrate different sensory systems to individually recognize both conspecifics and humans (cross-modal recognition, [8–10]) and to combine different facial cues of conspecifics to gather information on the environment [11]. Moreover, horses can also communicate their emotions [12] and understand facial expressions of both horses [13] and humans [14]. Finally, they reconcile after conflicts and engage in triadic post-conflict reunion to maintain the social homeostasis [15].

Taken together, these findings are indicative that horses, like other highly cognitive social animals, show some degree of awareness, which implies the ability to assess and deduce the significance of a situation according to both the social environment and the self [16].

Mirrors have been used to investigate a cognitive capacity that has been demonstrated only in a handful of species, including our own: the capacity of Self-Recognition, which is considered as a building-block of self-awareness. Mirror Self-Recognition (MSR) is not important *per se* but its importance resides in what it may unveil about the sense of self experienced by animals in relation to their social environment [17–19]. In short, MSR provides information about the cognitive and emotional skills that are necessary to develop complex social relationships [20] and to engage in behaviors relying on different levels of empathy [18,21,22].

The paradigm that has been used to verify MSR is based on the mark test, which was designed by [23] on chimpanzees. By placing a visible colored mark on the animal's body, it is possible to test whether a subject recognizes itself in the mirror. The mark must be placed on an out-of-view body part that is impossible to perceive without the help of a mirror. If the animal considers the image as its own, it will use its reflection in the mirror to detect the mark and will try to inspect, touch, explore or scrap it.

The MSR has been applied to humans [24], chimpanzees [23,25], orangutans [26], bonobos [27], and gorillas [28], while for monkeys there is no clear evidence of self-recognition [29,30]. Outside the primate order, elephants [31], dolphins [32] and magpies [33] seem to be able to recognize themselves in the mirror. All these species are characterized by high cognitive abilities, sophisticated neural capacities and a complex sociality, even though such skills do not seem to be sufficient to justify the presence of MSR; in fact, data on non-primate species come from a low number of tested subjects and are waiting to be replicated [18,19].

Horses have never been tested with the mirror mark-test paradigm so far, although mirrors have been advocated as beneficial sources of enrichment to improve the social environment of single housed subjects [34–36]. In domestic species it has been suggested that the use of mirrors reduces stereotyped behaviors and isolation stress [37–39]. However, the mirror provides only stimulation relying on visual cues to animals thus excluding all the other sensory inputs (auditory, olfactory, and tactile) that are usually combined to gain information on the social environment [40]. Accordingly, recent findings indicate that the simulation of social housing by mirrors is not sufficient to produce beneficial outcomes, because animals seem to be able to discriminate between a real social companion and its simple image [40;41].

The aim of this ethological study is to try to understand if horses show any evidence of MSR. To answer this question, we applied a mark test specifically designed for horses, which included two tactile control conditions.

## Materials and methods

### Ethics statements

This study was carried out in accordance with the recommendations of the Italian Animal Care Act (Decree Law 26/2014). The Ethical Committee on Animal Experimentation of the University of Pisa approved the experimental protocol (ref. n. 62131). The Italian Horse Protection gave consent to the use of their horses in this experiment.

### The horses

The study was conducted in October 2014 at the Italian Horse Protection rescue center (hosted at Villa Filicaja farm, Montaione, Tuscany, Italy). The tested animals were housed in a wide paddock (about 12 hectares) with *ad libitum* access to grass, hay and water. The horses had been living in a stable social group (10 horses) under naturalistic conditions for at least five years. Social deprivation has a strong impact on the psychological development in horses that can show behavioral signs of depression [42] and learned helplessness [43] as a consequence of housing and previous negative interactions with humans. For this reason, we decided to select free-ranging horses living in stable social groups.

Within the group, the selection of the horses was based on an interview of the caretaker following the questionnaire formulated by Momozawa et al. [44]. Specifically, we were interested in horses showing a high level of familiarity towards people and confidence with the stable in which the test would be performed, a tendency to adaptation to unfamiliar objects, and curiosity. Through preliminary observations we selected the pairs of horses characterized by a high level of affiliation and social proximity (see Table 1 for details), then the four horses were stabled in a paddock close to the testing arena with food and water available *ad libitum*.

**Table 1:** Description of the tested horses clustered in two groups according to their bonds (Pair 1: Gina/Calippo; Pair 2: Julia/Betsie). In addition to the information regarding sex, age, breed, test timing, we provided the color of the fur and the relative color of the mark (yellow or blue) that we selected in order to be clearly visible by both experimenters and tested horses.

	Name	Sex	Age	Breed	Hour of the test	Coat color	Mark color
**Pair 1**	Gina	female	10 years	Mixed breed	11.00 am	Grey	Blue
	Calippo	gelding	21 years	Italian saddle	12.00 am	Sorrel	Yellow
**Pair 2**	Julia	female	24 years	Budjonny	03.00 pm	Sorrel	Yellow
	Betsie	female	22years	Quarter horse	04.00 pm	Roan	Blue

### The testing arena

**Fig 1:**
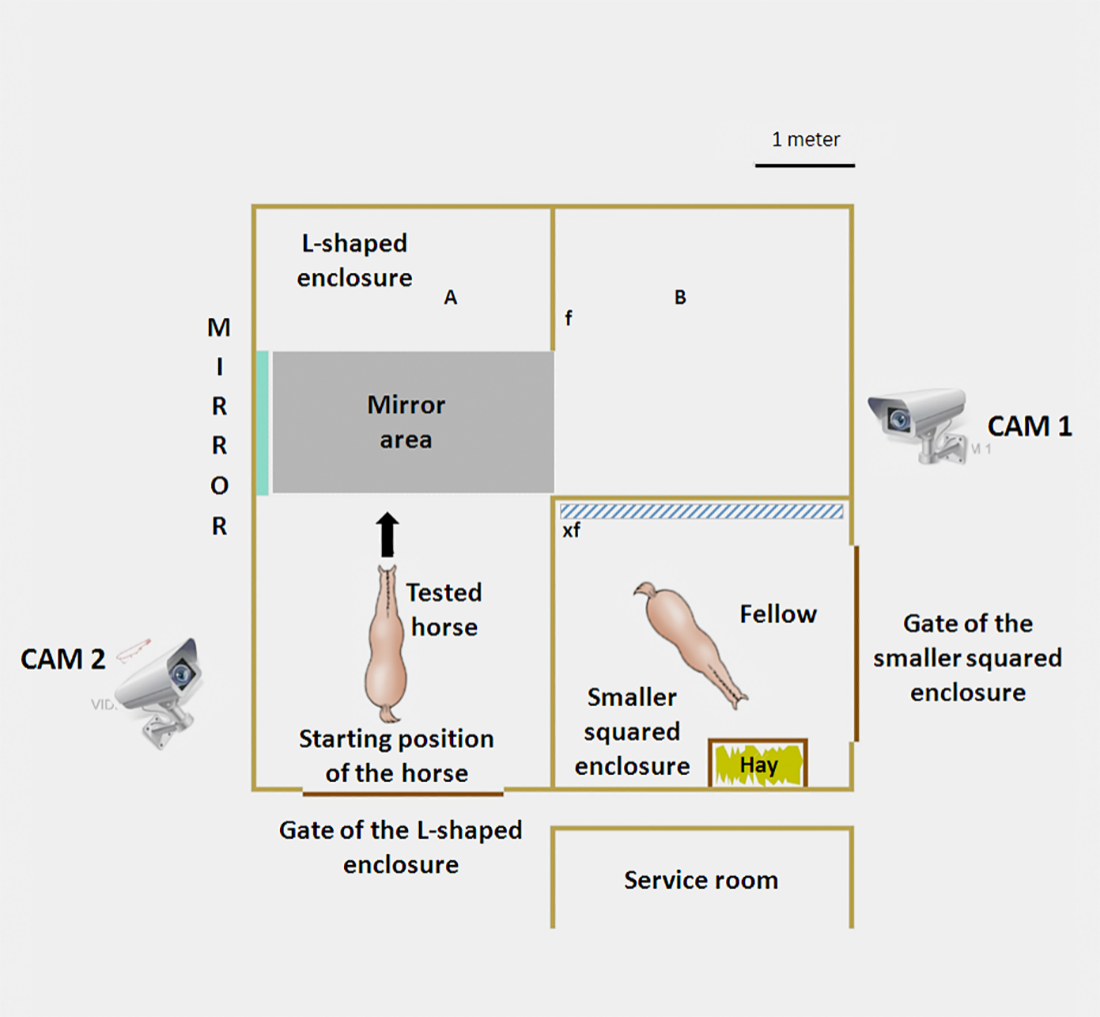
Detailed description of the testing area. A: the area of the L-shaped enclosure in which mirror was positioned; B: the other part of the L-shaped enclosure; f: the short fence delimiting the A and B areas; xf: the thick fence added to prevent the fellow horse looked at the mirror by leaning over the fence.

The testing arena consisted in a covered stable, composed by a main L-shaped enclosure (27 m^2^) and a smaller squared enclosure (9 m^2^). The two enclosures were separated by a wooden fence of about 1.20 meter high. The L-shaped enclosure was divided into two areas (A and B in Fig 1) connected by a passage formed by a 1.5 meter long fence (f in the Fig 1).

The fence separating the smaller squared area and the B area of the L-shaped fence was about 40 cm thick (xf in the Fig 1) to prevent the friend horse to accidentally see itself in the mirror by leaning over the fence.

The mirror consisted in a plexiglass panel (150 x 220 x 0.5cm). The dimension of the mirror was chosen on the basis of the real dimensions of the tested animals. The mirror was placed and glued on a wooden support and fixed to the external fence with plastic cable ties. It was placed on the left side of the A area in front of the passage between A and B areas and the “mirror area” was then defined as shown in Fig 1.

Two video cameras were positioned at about 3 meters of height to record the whole L-shaped enclosure. One camera (Cam 1—VisorTech^®^ ProfiWireless Micro-Webcam) was positioned in front of the mirror on the right side of the B area, while the other (Cam 2—Panasonic^®^ HDC-SD9) was placed on the left side of the A area close to the gate (see Fig 1).

### Experimental design

We tested one horse per time. To perform the experiment, one horse was conducted to the starting point positioned in the A area of the L-shaped enclosure (Fig 1). It was then let free after the halter removal. Concurrently, the friend horse was introduced into the smaller squared area, so that the two horses could interact only through the fence. The spatial proximity of the two horses prevented the potential negative emotional reaction linked to social deprivation which is particularly strong in subjects living in social groups [45]. During the experiment, the tested horse could freely move within the L-shaped enclosure and when passing from A to B area and *viceversa*, the horse had to enter the "mirror area".

When the test was over, the location of the two horses was interchanged, so that the friend horse could be tested. Each test lasted 1 hour and began when the head of the horse entered in the mirror area for the first time. Each horse was tested at the same time on consecutive days (Table 1) to compare the behavior of the tested horse across four different conditions (Covered Mirror, Open Mirror, Sham and Mark). The Mark condition was divided into MARK 1 and MARK 2 depending on the position of the colored mark, which were performed on subsequent days.

**Fig 2:**
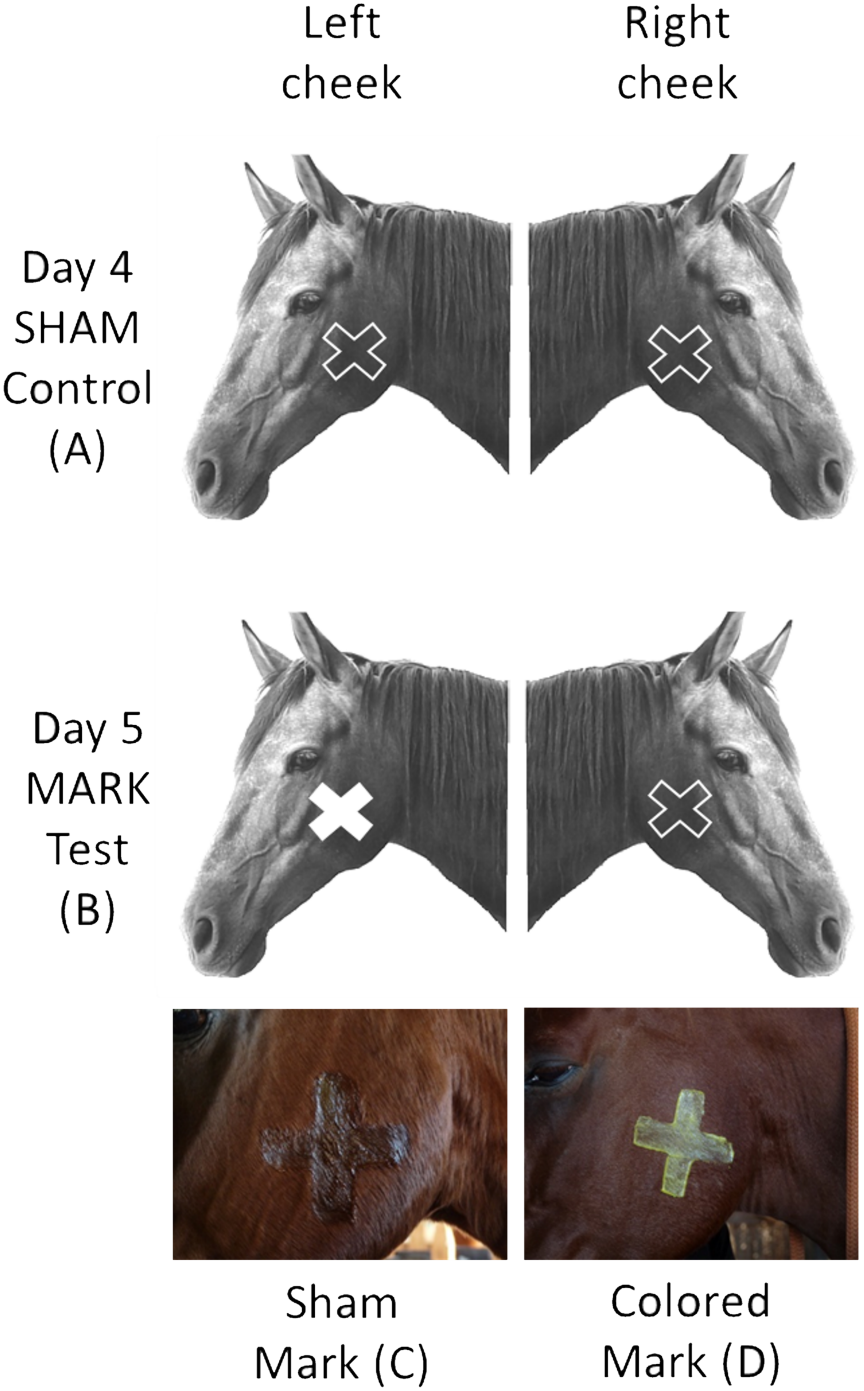
Shape, color and position of the sham and colored marks during the Sham and Mark test. Day 4 (SHAM control): both cheeks of the horse were painted with the sham mark (basal control for tactile cues) (A and C). Day 5 (MARK test 1): the left cheek was painted with colored mark (B and D, yellow for Julia and Calippo, blue for Betsie and Gina) while right cheek was painted with sham mark (C). Day 6 (MARK test 2): the position of sham and colored marks was inverted and, therefore, colored mark was positioned on the right side while sham mark was on the left side. The presence of the sham mark on the opposite cheek of the colored mark (during MARK test 1 and 2), was necessary to avoid the bias due to a tactile stimulation.

Although all horses were accustomed to the stable, we started with a familiarization condition during which the tested horse spent one hour in the L-shaped enclosure in absence of the mirror (day 1). This phase was necessary to exclude the presence of aberrant or stress-related behaviors. In the second condition (Covered Mirror, CM) the mirror was present but positioned with the reflecting surface facing outwards the L-shaped enclosure (day 2). In the third condition (Open Mirror, OM) the reflecting surface of the mirror was facing towards the L-shaped enclosure, so that the tested horse could perceive its image in the mirror (day 3) (Fig 1). For the following tests the mirror was left in the same position as day 3. On day 4 (SHAM), a transparent cross-shaped drawing (10 cm) was applied on both cheeks of the tested horse (control, Fig 2A and 2C). The transparent cross-shaped drawing consisted in ultrasound water gel (Ultrasound gel, Gima, Milan). This was necessary to control for the possibility that the animal’s behavior was driven by the tactile sensation of the mark rather than the visual mark itself [32]. On day 5 (MARK 1), the transparent cross-shaped drawing was applied again only on the right cheek to avoid touching bias (Fig 2B). The cross-shaped drawing on the left side was colored by adding a yellow or blue odorless, hypoallergenic eye-shadow powder to the transparent ultrasound water gel (Fig 2D, Table 1). On day 6 (MARK 2) the position of sham and colored marks was inverted and therefore, colored mark was positioned on the right side while sham mark was on the left side.

Due to the asymmetric structure of the L-shaped enclosure and the starting position of the horse, the left side of the head was the first to come into view in the mirror. By following this procedure, we made sure that the horse could perceive the colored mark even if it passed through the mirror area only once. For that reason the colored mark in MARK 1 was placed on the left cheek. This procedure creates a bias in the MARK 2 (when colored mark was positioned on the right cheek) and this could provide information about the influence of the first exposure of the colored mark (MARK 1) on the subsequent task (MARK 2).

The selection of two primary colors (yellow or blue) to mark the cheek of the horse was based on horse color perception [46]. To maximize the chromatic contrast and increase the likelihood that the tested horse could actually perceive the colored mark as different from the transparent one, we selected blue or yellow eye-shadow powder as a function of coat color (Table 1). The transparent cross-shaped drawing controlled for both olfactory and tactile cues (i.e., texture), leaving only the visual component to differentiate between the colored mark and the transparent mark. The mark was placed on the cheek because the panoramic visual field of horses does not cover this head area [47] and, therefore, the mark could be detected by the tested horse only with the guidance of the mirror. The choice to arrange the mark on the cheek relied on the easiness for the horse to reach that area by the limbs.

Fifteen minutes before the test, the caretaker applied the mark (sham, yellow or blue) during a 10-min grooming session performed on the whole body of the horse to exclude the possibility that the horse was aware of being marked [19]. Concurrently, a repellent substance (Tri Tec, Chifa srl, Angera, VA) was applied on the whole body of the horse to avoid insect disturbance. During the test nobody was present in the testing area. Immediately after the release of the horse the experimenters and caretakers moved into the service room where they had the possibility to control the progress of the test by the remote control of Cam 1.

### Behavioral definitions and data analysis

Under CM and OM conditions we recorded the following behavioral patterns of the tested horse: staying in front of the mirror (FRONT), exploring the mirror (EXP), looking behind (LOOK) and opening the mouth and protruding the tongue in front of the mirror (MOUTH). During the four experimental conditions (CM, OM, SHAM and MARK) we also recorded the scraping behavior toward cheeks (SCRA). See Table 2 for the description of the behaviors.

**Table 2:** Description of the behaviors performed by the horses once entered in the “mirror area”. Attention to and exploration of the mirror, looking behind the mirror and mouth movements were collected under Open (OM) and Covered Mirror (CM) conditions; Scraping events were collected under the four experimental conditions (OM, CM, SHAM and MARK). Videos are provided in Supporting Materials showing the horses performing such behaviors.

Description	Data collected	Supporting information
**Attention to the mirror (FRONT)**		
The head of the tested horse had to be in the mirror area with one or both eyes visible in the reflected image for at least 3 seconds.	The time (secs) spent performing FRONT (state behavior)	S1 Video S2 Video
Exploration of the mirror (EXP)*		
The behavior EXP included the time (secs) spent by the tested horse sniffing, licking and touching the mirror with the mouth.	The time (secs) spent performing EXP (state behavior)	S3 Video
**Looking behind the mirror (LOOK)**		
When the horse was close to the mirror (<1m) and put its head and neck forward the fence. We classified the behavior as a LOOK event only when the horse turned its head toward the rear side of the mirror thus using its visual binocular field.	The number of times the horse performs LOOK behavior (event behavior)	S4 Video
**Movements of the mouth (MOUTH)**		
The behavior MOUTH included each mouth opening and tongue protrusion only when these behaviors were performed in FRONT of the mirror	The number of times the horse performs MOUTH behavior (event behavior)	S5 Video
**Scraping (SCRA)**		
The horse rubbed the lateral side of its head (area between the labial and the caudal part of the mandibular branch) with both the ipsi- and contra-lateral forelimb. Other self-directed behaviors performed towards the muzzle or the ears were excluded. Moreover, during the SCRA event the horse could also rub the lateral side of its head against the surface of a fence.	The number of times the horse performs SCRA behavior (event behavior). Two SCRA events were considered as distinct when the horse separated its head from the paw or from the fence.	S1 Fig S6 Video S7 Video

* Sniffing, licking and touching were included in a single behavioral category as it is not possible to keep the different sensory modalities separated (e.g., licking includes both touching and sniffing)

The video analysis was performed independently by two observers who reached a high reliability index for each behavioral item considered (Cohen's kappa ≥ 0.90). The frequency of each behavior (FRONT, EXP, LOOK and MOUTH) performed in the CM and the OM conditions were compared by the Chi-Square "Goodness of Fit" test. The same test was used to compare the frequency of SCRA behavior across the four conditions (CM, OM, SHAM and MARK). Furthermore, the frequency of SCRA behavior between the SHAM and MARK conditions was compared by using the two-tailed Exact Binomial test. Statistical analyses were performed via the VassarStats website (http://vassarstats.net/).

## Results

### Individual behavior towards the mirror: Covered and Open Mirror conditions

To assess whether the reflecting surface of the mirror rather than the novelty of the apparatus induced a changing in the behavior of horses, we explored the activity towards the mirror that the horses performed in presence of the covered (CM) and open mirror (OM). Under CM condition there was no remarkable behavior in front of the covered mirror in any of the tested horses. On the contrary, under OM condition the behavior of the horses strongly differed. All of the tested horses spent a significantly higher amount of time in FRONT of the open compared to the covered mirror. Three horses out of four engaged in a significantly longer exploring activity (EXP) under OM than CM. Furthermore, all horses looked behind (LOOK) the mirror only when it was uncovered. In the OM, three out of four horses also performed MOUTH movements (mouth opening and tongue protrusion) when they were in front of the reflecting surface with one horse reaching statistical significance (Table 3).

**Table 3:** The table shows the amount of time (seconds) that each horse spent in front of and exploring the mirror in Open Mirror (OM) and Covered Mirror (CM) conditions. In the table, the number of the times the horse looked behind the mirror and all the mouth patterns the animal performed in front of the mirror are also reported for both conditions, OM and CM. Under OM condition all the horses spent a higher amount of time in front of the mirror and three horses out of four engaged in a significantly longer time exploring it, compared to CM condition. All of the tested horses looked behind the mirror in OM condition and three out of four horses performed mouth movements when they were in front of the reflecting surface.

	Time (secs) in front of the mirror			Time (secs) exploring the mirror			Looking behind		Mouth opening/tongue protrusion in front of the mirror		
Subject	OM	CM	Chi-square	OM	CM	Chi-square	OM	CM	OM	CM	Chi-square
Betsie	118	68	12.9 (p = 0.0003)	0	3	n.d.	3	0	0	0	n.d.
Calippo	187	5	170.6 (p < .0001)	42	0	40.0 (p < .0001)	2	0	16	3	7.6 (p = 0.0059)
Gina	228	61	95.3 (p < .0001)	57	7	37.5 (p < .0001)	4	0	2	0	n.d.
Julia	1236	141	869.2 (p < .0001)	50	0	48.0 (p < .0001)	6	0	11	6	0.9 (p = 0.3323)

### Self-directed behaviors under the four conditions: CM, OM, SHAM and MARK

**Fig 3:**
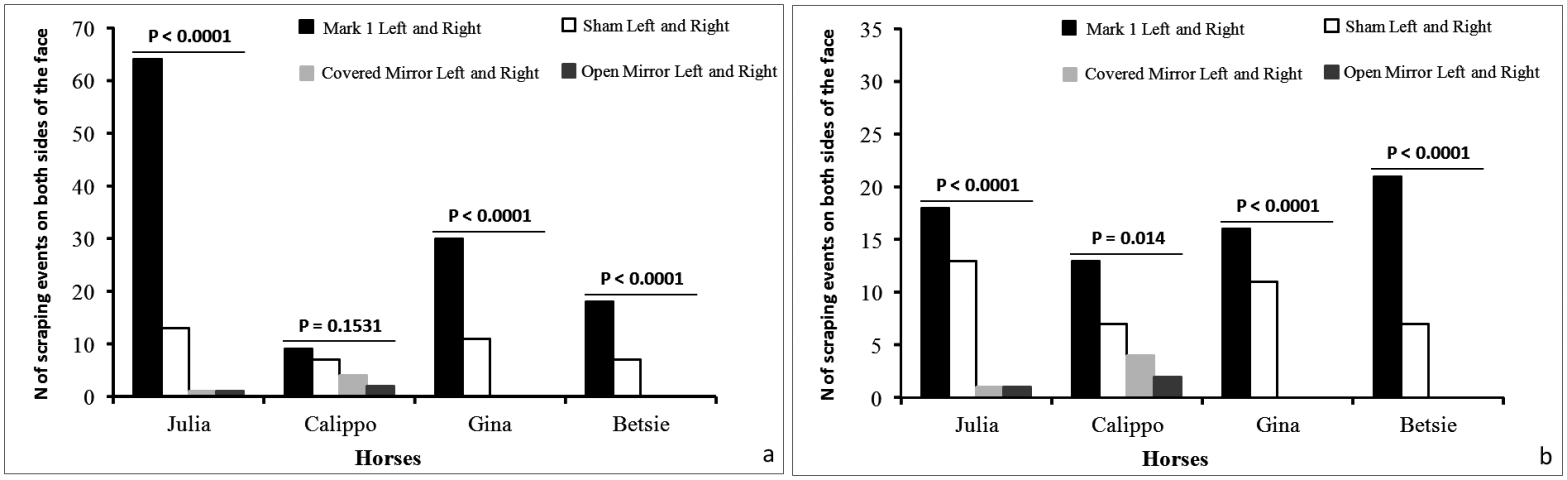
Frequency of scraping events directed towards both cheeks. Bar-graphs showing the frequency of the scraping events (SCRA) that each subject directed towards both sides of their face (see S1 Fig) across the four different conditions when the colored mark was positioned on the left cheek **(a)** (CM, OM, SHAM, MARK 1; df = 3; Julia: χ^2^ = 137.05, p < 0.0001; Calippo: χ^2^ = 5.27, p = 0.1531; Gina: χ^2^ = 58.61, p < 0.0001; Betsie: χ^2^ = 34.68, p < 0.0001) and when it was placed on the right cheek **(b)** (CM, OM, SHAM, MARK 2; df = 3; Julia: χ^2^ = 27, p < 0.0001; Calippo: χ^2^ = 10.62, p = 0.014; Gina: χ^2^ = 28.85, p < 0.0001; Betsie: χ^2^ = 42, p < 0.0001).

Betsie, Gina and Julia performed SCRA behavior towards both cheeks with a significantly different frequency across the four conditions: CM, OM, SHAM and MARK 1 (Fig 3A) and showed a significant increase in the SCRA activity towards both cheeks in the MARK 1 compared to the SHAM condition (Table 4).

**Table 4:** Comparison (Chi-square and Binomial tests) between the amount of scraping (SCRA) behaviors in MARK and SHAM conditions: The frequency of SCRA behavior towards both left and right cheeks is reported in the two conditions (SHAM and MARK 1, 2). There was a significant increase of SCRA towards both sides of the face under MARK 1 condition compared to SHAM condition in three out of four horses. One out of four horses performed SCRA behavior at higher frequency in MARK 2 compared to SHAM condition. The table also shows the SCRA activity performed only towards the left cheek which corresponded to the mark side (MARK 1, left), with the same three horses showing a significant increase of SCRA in MARK 1 compared to the SHAM condition (left). Regarding the SCRA events towards the right cheek (MARK 2, right), there was no difference in SCRA directed to the right cheek between MARK 2 (right) and SHAM condition (right).

	MARK 1 (left and right)*	SHAM (left and right)*	Chi Square (Binomial test)^§^
Julia	64	13	p < 0.0001; χ^2^ = 32.46 (p < 0.0001; z = 5.7)
Calippo	9	7	p = 0.8065; χ^2^ = 0.06 (p = 0.8036; z = 0.25)
Gina	30	11	p = 0.0049; χ^2^ = 7.9 (p = 0.0043; z = 2.81)
Betsie	18	7	p = 0.0455; χ^2^ = 4 (p = 0.0433; z = 2)
	MARK 2 (left and right)*	SHAM (left and right)*	Chi Square (Binomial test)^§^
Julia	18	13	p = 0.4708; χ^2^ = 0.52 (p = 0.4731; z = 0.72)
Calippo	13	7	p = 0.2636; χ^2^ = 1.25 (p = 0.2632; z = 1.12)
Gina	16	11	p = 0.4386; χ^2^ = 0.6 (p = 0.4421; z = 0.77)
Betsie	21	7	p = 0.014; χ^2^ = 6.04 (p = 0.0125; z = 2.46)
	MARK 1 (left)^#^	SHAM (left)^#^	Chi Square (Binomial test)^§^
Julia	41	8	p < 0.0001; χ^2^ = 20.9 (p < 0.0001; z = 4.57)
Calippo	6	6	p = 1; χ^2^ = 0 (p = 1; z = 0)
Gina	16	5	p = 0.0291; χ^2^ = 4.76 (p = 0.0266; z = 2.18)
Betsie	8	1	p = 0.0455; χ^2^ = 4 (p = 0.0391; z = n.a.)
	MARK 2 (right)^#^	SHAM (right)^#^	Chi Square (Binomial test)^§^
Julia	8	5	p = 0.5839; χ^2^ = 0.3 (p = 0.5811; z = 0.55)
Calippo	6	1	p = 0.1311; χ^2^ = 2.28 (p = 0.125; z = n.a.)
Gina	6	6	p = 1; χ^2^ = 0 (p = 1; z = 0)
Betsie	8	6	p = 0.7773; χ^2^ = 0.08 (p = 0.7905; z = 0.27)

* The total of SCRA behaviors directed to both cheeks of the face (left + right) in MARK 1 vs SHAM and MARK 2 vs SHAM

^#^ The total of SCRA behaviors directed to the left cheek (MARK 1 vs SHAM left) or the right cheek (MARK 2 vs SHAM right)

^§^ Chi-Square "Goodness of Fit" Test and Binomial test (between brackets). Binomial test has been included because two comparisons have expected frequencies lower than 5.0

In the MARK 2 all horses performed SCRA behavior towards both cheeks with a high significantly different frequency across the four conditions: CM, OM, SHAM and MARK 2 (Fig 3B). Moreover, Betsie performed SCRA behavior with significantly higher frequency on both cheeks in MARK 2 than in SHAM condition (Table 4).

**Fig 4:**
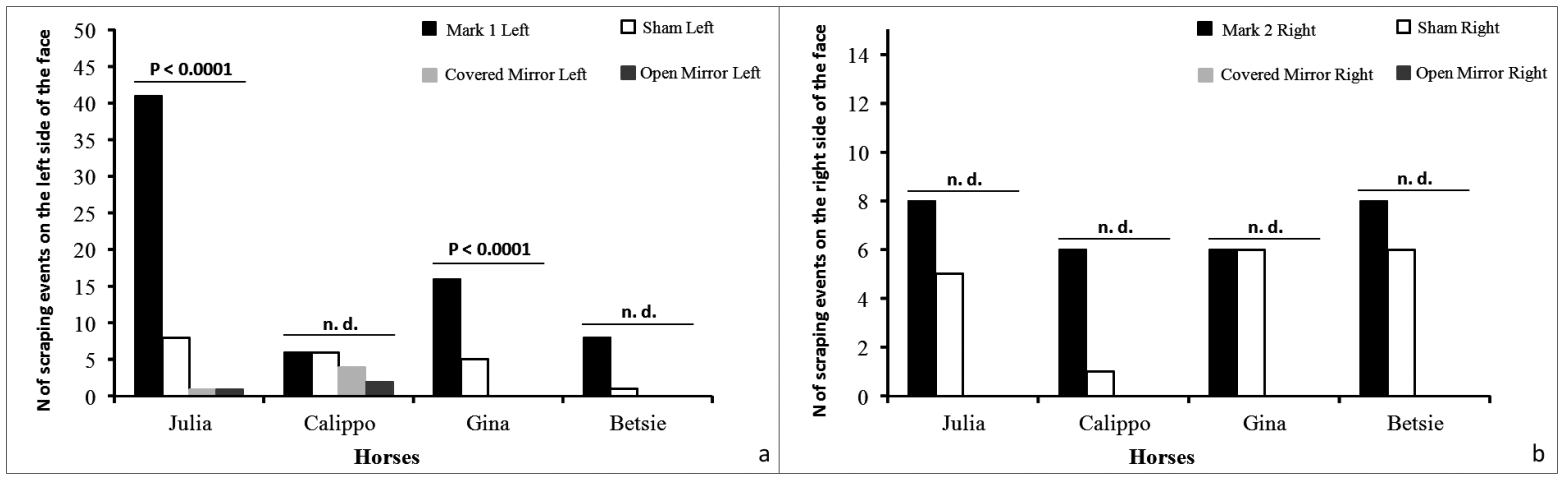
Frequency of scraping events directed towards the left or the right cheek. Bar-graphs showing the frequency of the scraping events (SCRA) that each subject directed specifically towards the left side of their face (see S1 Fig) across the four different conditions when the colored mark was placed on the left cheek **(a)** (CM, OM, SHAM, MARK 1; df = 3; Julia: χ^2^ = 86.02, p < 0.0001; Calippo: nd—expected values less than 5; Gina: χ^2^ = 32.52, p < 0.0001; Betsie: nd—expected values less than 5) and when it was placed on the right cheek **(b)** (CM, OM, SHAM, MARK 2; df = 3; Julia, Calippo, Gina, Betsie: nd—expected values less than 5).

As for the behavior specifically directed towards the left side of the head in the MARK 1 and SHAM conditions, we were able to test the frequency distribution between the four conditions (CM, OM, SHAM and MARK) of the SCRA behavior only for two subjects (Gina and Julia) because for the other two subjects the expected frequency was less than five. Gina and Julia maintained a strong difference in the distribution of SCRA across the four conditions (Fig 4A). Moreover, the binomial and chi-square tests indicate that Betsie, Gina and Julia engaged in SCRA activity towards the left cheek at a higher frequency in the MARK compared to the SHAM condition (Table 4).

Regarding the behavior specifically directed towards the right cheeks, it was not possible to test the SCRA levels across the four conditions (CM, OM, SHAM and MARK) because the expected frequency was less than five (Fig 4B). Statistics indicates that there was no difference in SCRA activity towards the right cheek between MARK 2 and SHAM condition (Table 4). All relevant data are included (S1 and S2 Datasets).

## Discussion

The horses tested in our pilot study showed some behavioral patterns in response to the presence of the reflecting surface which were not performed in the other conditions. Therefore, differences in the attentional behavior toward the mirror are ascribable to the presence of the reflecting surface rather than to the object itself. Despite such behaviors are ambiguous, at least some of them indicate that horses could understand that the image in the mirror is not a real animal, as it is evident in other mammalian species [41]. The increase in the attentional and exploratory behaviors towards the reflecting surface might be explained by the violation of expectancy paradigm [48–50], by which it has been demonstrated that horses are able to associate multiple cues to individually recognize conspecifics and people (cross-modal recognition, [8,9]). In our case, the image in the mirror met the visual criterion, but it did not provide the expected tactile and olfactory information. Hence, the image of the horse could violate the spontaneous association of multiple cues because the pieces of information collected in front of the mirror are incongruent. Even though the violation of expectancy paradigm has never been used to interpret the increase of attentional and exploratory behaviors during the first phases of MSR studies, we suggest that it could be a possible explanation.

To successfully pass the mark test, a sequence of behavioral steps towards the mirror must be performed [51], which is indicative of the cognitive process that leads animals to understand that the image in the mirror is the image of self [31].

Specifically, in the first step the animal should perform a social response (often agonistic behaviors) toward the image [51]. Only one of the tested horses showed unambiguous agonistic behaviors toward the image in the mirror. The second step is related to the mirror physical inspection [51], including looking behind the mirror. Compared to the covered mirror, in the open mirror condition the four horses spent much more time in the mirror area and three of them explored the mirror for a longer time. Looking behind the mirror has been reported in several primate and non-primate species [23,31,33,52,53] and it has been interpreted as the attempt to check for the actual absence/presence of the conspecific behind the reflecting surface. All of our tested horses looked behind the mirror several times (Table 2), while none of them did it in the covered mirror condition. This can be explained by the necessity of the tested horses to solve the incongruity of the perceived cues collected during their FRONT and EXP behaviors [48].

During the third step, animals should perform repetitive mirror-testing behaviors (i.e. inspection of body parts that are visible only through the mirror) that represent the attempt of testing if the mirrored image is the image of self [31,51]. As third step, our horses showed ambiguous behaviors and three of them (Calippo, Gina and Julia) opened their mouths and protruded their tongues (MOUTH) more frequently in Open Mirror than in Covered Mirror condition, but only one (Calippo) reached statistical significance (Table 3). The mouth movements could be interpreted as a vacuum chewing (chewing with nothing in the mouth, [54]), which may be related to frustration linked to the testing condition or to the impossibility to reach the seen conspecific.

The paradigm of the MSR endorses that animals should overcome all the three steps to access to the "mark phase" [51] which represents the final step. This step is passed if a subject spontaneously uses the mirror to touch an otherwise imperceptible mark on its own body [23,51]. Considering our previous results, the tested horses could not be admitted to the mark test, as they did not show the requested sequence of behaviors in the first three steps that are considered as mandatory by some scholars. However, this procedure is still highly debated and it has not been applied in some studies [20–22]. Moreover, Povinelli et al. [25] found that 11% of chimpanzees that did not pass the first three steps—and therefore were classified as negative or ambiguous cases—passed the mark test successfully. This result suggests that this procedure can produce false negatives.

The behavioral patterns the horses showed in the Open Mirror condition (FRONT, EXP, LOOK and MOUTH) were not always performed in the same order, even though FRONT and EXP always preceded LOOK and MOUTH (see S8 Video). Therefore, despite the series of the behavioral patterns in the three steps was not performed as expected, our horses showed a specific sequence of behavior in the Open Mirror compared to the Covered Mirror condition, thus indicating the motivation to explore the image in the mirror. Due to the open-ended nature of our pilot study and the ambiguous responses given by our horses during the first three steps, we decided to admit all of them to the mark test anyway.

The comparison of the scraping events (SCRA) performed towards both cheeks across the four conditions (Fig 3), provides information about the influence of the mark (colored or sham) on self-directed behaviors. The presence of both sham and colored marks actually affected the frequency of SCRA towards both cheeks, thus indicating that horses are able to perceive the mark. This result *per se*, however, does not permit to differentiate the two sensory components of the mark (tactile *vs* visual). In presence of a colored mark (MARK 1: sham dx and mark sx), two out of four horses significantly differed in scraping their colored-mark cheek across the four conditions (Fig 4A). Unfortunately, as it occurred in other studies on MSR [31–33], the amount of SCRA recorded in all the four conditions for MARK 2 did not permit to test for possible statistical differences (expected values <5), although the behaviors seemed to differ across the conditions (Fig 4B).

The direct comparison of the SCRA events between mark and sham conditions provides indication about the role of tactile and visual components in the perception of the stimulus. In one horse (Betsie) the amount of SCRA was always significantly higher in presence of the colored mark (in both MARK 1 and 2) compared with the sham mark, while in other two horses (Julia and Gina) such difference was present only in MARK 1 (Table 3). In three horses the frequency of SCRA directed to the left colored cheek was statistically higher than SCRA performed towards the transparent mark on the same side (MARK 1 vs SHAM, Table 3) thus suggesting that the visual cue elicits SCRA behavior in front of the mirror more than the tactile one. The ambiguous outcome obtained in MARK 2 (colored mark on the right cheek) can be mainly explained by the bias due to the starting position of the horse in respect to the reflecting surface. From this starting position the right side cannot be seen at the first passage across the "mirror area" (Fig 1). A wide bulk of literature indicates that a selective attention is mainly influenced by the regularities of the tasks, providing learning effects. These functional adjustments of the attentional mechanism may occur on a trial-by-trial basis in the short term period, hence indicating that the setting of attentional priority is affected by the evaluation of the preceding events (for an extensive review see [55]). Therefore, we cannot exclude that the bias toward the left side induced by our experimental design could affect the behavior of the horses in the subsequent MARK 2 condition. Even if horses could perceive the colored mark positioned on the right cheek, their selective attention could be mainly focused on the left cheek due to their previous experience (MARK 1), thus paying less attention to the right side. The lower frequency of SCRA on the right cheek (MARK 2) could be also due by the hemispheric laterality in social information processing in animals (for an extensive review see [56]) that has been highlighted also in horses [57,58]. To better understand such attentional bias effect, the mark test sequence should be carefully evaluated in future studies.

Another possible criticism of the mark test paradigm could be linked to the species-specific peculiarity of the stimulus detection that cannot completely exclude the perception of the control mark. For example, one of the tested horses (Calippo) scraped its left cheek the same amount of times in both SHAM and MARK 1. Depending on the different inclination of his head, Calippo could actually see the brightness of the sham mark reflecting the light (see S9 Video for details). The ability of horses to better perceive the brightness of objects could be linked to the *tapetum lucidum*, an intraocular reflecting structure which is present also in other mammals (such as elephants and dolphins), but not in monkeys and apes [59]. A similar methodological bias has also been reported for magpies [33], because birds might perceive the tactile component of the sham stickers [60]. This explains the difficulty to create a perfectly "invisible" mark to be used as a neutral stimulus to control for the possible bias generated by the tactile component of the mark in non-primate species. Therefore, in our study the impossibility to completely exclude the visual cue of the sham mark probably increased the possibility to make a type II error (i.e., false negative). Reading our results with other studies, it seems that an interesting question arises: is the current paradigm of mirror self-recognition suitable for all species? In order to obtain clearer evidence about MSR in horses it would be necessary to adapt the experimental design of mark-test to this species by taking into account its cognitive, social and perceptual peculiarities.

In conclusion, although horses are capable of a global, integrated, multisensory representation of specific individuals in intra- and inter-specific social exchanges [8,11], our horses did not match the complete expected behavioral steps to fit the mirror self-recognition paradigm. Without replication of data, the self-directed behavior towards the colored mark showed by three horses in Mark 1 and one horse in Mark 1 and 2 is not sufficient *per se* to affirm that horses are capable of mirror self-recognition.

## Supporting information

S1 Fig The area of scraping. Red dots delimit the area of the scraping (SCRA) behavior.
(TIF)

S1 Video Betsie approaches the mirror frontally. At 00.00 she comes from the B area of the L-shaped paddock and stops within the “mirror” area (from 00.02) to look her image.
(AVI)

S2 Video Julia approaches the mirror laterally. Since she has just been released from the starting position, Julia enters the “mirror” area from the left side (at 00.01 she turns her head towards the mirror). From 00.02 to 00.07 she stares at her image reflected on the surface.
(AVI)

S3 Video Calippo explores the surface of the mirror. He sniffs the mirror producing vapor traces on the reflecting surface while breathing very close to it (from 00.02 to 00.12). Calippo replicates the same behavior at 00.16 (from 00.16 to 00.22).
(AVI)

S4 Video Julia approaches the mirror for the first time. Julia is seeing herself reflected in it. Firstly, she shows hesitation (at 00.01 she withdraws); after, at 00.07, she gets close to the mirror, she turned her head towards the mirror and stares at her image (at 00.10 and again at 00.13). Later, Julia quickly moves to look behind the mirror (00.19).
(AVI)

S5 Video Calippo protrudes its tongue in front of the mirror repeatedly. Tongue protrusion (from 00.03 to 00.15) is probably a repetitive testing behavior to check the movement of the reflecting image.
(AVI)

S6 Video Julia scrapes the left cheek. Julia scraps the left cheek with the yellow mark on her left foreleg (at 00.05) (the mark is visible in the reflecting image). Later, Julia gets close to the mirror (at 00.07), stares at it (at 00.13) and breathes close to it (00.15). At 00.19 she scraps again the left cheek on her right foreleg.
(AVI)

S7 Video Gina scraps the blue mark with the foreleg and the fence. Gina firstly scraps the blue marked cheek on her left foreleg (at 00.01). Then, she scraps the same marked cheek (left) towards the fence (at 00.10).
(AVI)

S8 Video The beginning of the mirror exposure. At the beginning of the mirror exposure, the horses often performed behaviors such as staying in front of the mirror, exploring it and looking behind it in a very quickly sequence, as showed in this video. Gina looks towards the mirror from the left side of the “mirror” area (from 00.00), then she approaches the mirror (at 00.15), sniffing its surface (from 00.20 to 01.02). A vapor trace is clearly visible (at 00.52). At 00.58, she touches the reflecting surface of the mirror. At 01.05 she turns around herself to look behind the mirror (01.11).
(AVI)

S9 Video Calippo performed the SCRA behavior even directed to the sham mark. This video refers to the test of day 4 when both cheeks of the horses were sham marked (SHAM). In this video Calippo clearly changes the inclination of his head (at 00.09) while staring at his left cheek reflected in the mirror. Then he scrapes his left cheek on his left foreleg two times (at 00.12 and at 00.15). Later, at 00.26 Calippo sniffs the mirror’s surface (a vapor ring is visible) and scrapes his left cheek two times (at 00.28 and at 00.34 on his right foreleg).
(AVI)

S1 Dataset Raw data related to no mirror (absence of the mirror), Covered Mirror and Open Mirror conditions. (XLSX)

S2 Dataset Raw data related to Sham and Mark (1 and 2) conditions. (XLSX)
